# Responses of leaf hydraulic traits of *Schoenoplectus tabernaemontani* to increasing temperature and CO_2_ concentrations

**DOI:** 10.1186/s40529-022-00331-2

**Published:** 2022-01-24

**Authors:** Yao Zhao, Mei Sun, Huijun Guo, Chunhui Feng, Zhenya Liu, Junping Xu

**Affiliations:** 1 Yunnan Key Laboratory of Plateau Wetland Conservation, Restoration and Ecological Services, Southwest Forestry University, Kunming, 650224 Yunnan China; 2 National Plateau Wetlands Research Center, Southwest Forestry University, Kunming, 650224 Yunnan China

**Keywords:** Wetland plant, Ecological response, Plateau wetlands, Climate change

## Abstract

**Background:**

Against the background of a changing climate, the responses of functional traits of plateau wetland plants to increasing temperatures and CO_2_ concentrations need to be understood. Hydraulic traits are the key for plants to maintain their ecological functions and affect their growth and survival. However, few studies have comprehensively considered the response strategies of wetland plants' hydraulic traits to environmental changes in the context of water and matter transport, loss, and retention. According to the latest IPCC prediction results, we performed experiments under increased temperature (2 °C) and CO_2_ levels (850 μmol/mol) in an artificial Sealed-top Chamber (STC) to investigate the responses of the hydraulic characteristics of *Schoenoplectus tabernaemontani*, the dominant species in plateau wetlands in China.

**Results:**

Compared with the CK group, net photosynthetic rate, transpiration rate, stomatal length, cuticle thickness, vascular bundle length, vascular bundle width, and vascular bundle area of *S. tabernaemontani* in the ET group were significantly reduced, whereas stomatal density and vein density increased significantly. Compared with the CK group, the hydraulic traits of *S. tabernaemontani* in the EC group were reduced considerably in stomatal length and cuticle thickness but increased dramatically in stomatal density, and there were no significant differences between other parameter values and the control group. Net photosynthetic rate was significantly positively correlated with stomatal length, cuticle thickness, and vascular bundle length, and stomatal conductance was significantly positively correlated with cuticle thickness. The transpiration rate was significantly positively correlated with cuticle thickness, epidermal cell area, vascular bundle length, vascular bundle width, and vascular bundle area. Regarding the hydraulic traits, there was a significant negative correlation between stomatal density and stomatal length, or cuticle thickness, and a significant positive correlation between the latter two. The epidermal cell area was significantly positively correlated with epidermal thickness, vascular bundle length, vascular bundle width, and vascular bundle area.

**Conclusions:**

Increased temperature and CO_2_ levels are not conducive to the photosynthetic activity of *S. tabernaemontani*. Photosynthetic rate, stomatal density and size, vein density, epidermal structure size, and vascular bundle size play an essential role in the adaptation of this species to changes in temperature and CO_2_ concentration. In the process of adaptation, hydraulic traits are not isolated from each other, and there is a functional association among traits. This study provide a scientific basis for the management and protection of plateau wetlands.

## Introduction

Climate warming and increasing CO_2_ concentrations are the two main characteristics of global climate change. According to the fifth IPCC global climate change assessment report, the global average surface air temperature at the end of this century will increase by 0.3–4.8 °C based on data from 1986 to 2005, and the concentration of CO_2_ in the atmosphere will reach 540–970 μmol·mol^−1^ (IPCC 2014). Many studies have shown that climate change, characterized by warming and rising CO_2_ concentrations, significantly affects the structure and function of the earth's ecosystems (Xia et al. 2020; Feng et al. 2020a, b).

Wetland ecosystems are unique habitats formed by land and water and play a vital role in maintaining biodiversity and providing ecosystem services (Li et al. 2018). However, they are highly vulnerable to climatic changes (Day et al. 2005). Plateau wetlands are an essential part of Chinese ecological environment. Because of their high elevation and specific terrain, they are extremely sensitive to climate change (Feng et al. 2019). Wetland plants, as the functional carriers of wetlands, show significant changes in their morphology, structure, and physiological functions under a changing climate, indicating adaptation to environmental changes (Zhao et al. 2009). In this context, studying the responses of the functional traits of plateau wetland plants to changing climate characteristics is an essential aspect of exploring and predicting change laws in wetland ecosystems and can facilitate the prediction of plateau wetland evolution and ecological function processes (Guittar et al. 2016).

Plant hydraulic traits are the general term for a class of functional traits that can significantly affect water transmission, loss, and retention, thereby affecting physiological functions such as photosynthetic productivity (Lawren et al. 2006). The stomata are the main channels for water vapor exchange between plants and the atmosphere, and their size and density changes directly affect the water loss rate and sensitivity of plants (Zuo et al. 2005). The cuticle, epidermal cells, and other epidermal structures are essential for plant water conservation (Li et al. 2016), and the vascular bundle traits play an important role in water transport and distribution in plants (Fang et al. 2003). Terrestrial plants are frequently constrained by water availability, and changes in hydraulic traits and functional regulation are the key to the growth, survival, and development of land plants. (Anderegg et al. 2016; Sack et al. 2016). Hydraulic traits are also the main parameters reflecting the functions of wetland plants such as erect state, resistance to mechanical damage, water vapor and material exchange, water and material transmission, and photosynthetic production, among others (Sack et al. 2016; María et al. 2013; Chen et al. 2009). In this sense, focusing on the adaptability of the hydraulic traits of wetland plants is crucial when exploring their ecological adaptation mechanisms, with consequent information on wetland protection. However, systematic research on the hydraulic traits of plateau wetland plants is still scarce.

This study used *S. tabernaemontani*, the dominant plant species of the plateau wetland in the Yunnan area, as the research object and applied the Sealed-top Chamber (STC) to simulate increases in temperature and CO_2_ concentration. By determining stomatal density and size, vein density, epidermal structure size, vascular bundle structure, photosynthetic gas exchange parameters, and other hydraulic traits, the responses of *S. tabernaemontani* to increasing temperature and CO_2_ levels were determined. This study provides a scientific basis for the management and protection of plateau wetlands.

## Materials and methods

### Research materials and experimental settings

*S. tabernaemontani* is widely distributed in the lakeside zone of plateau wetlands in the Yunnan area, China. The dominance of *S. tabernaemontani* at different elevations is discrepant, and the species is susceptible to changes in environmental factors. It shows solid ecological plasticity, making it an ideal material for studying the responses of functional traits to changes in environmental factors and to assess the adaptability of wetland plant functional traits to overall climatic changes.

In April 2015, healthy and evenly growing *S. tabernaemontani* specimens in the lakeside zone of Dianchi Lake were selected and transplanted into an experimental barrel with a diameter of 35 cm and a height of 25 cm. The cultivation substrate was in-situ soil from the lakeside zone of Dianchi Lake, with each plant receiving the same amount of soil. After 14 days of adaptation under natural conditions, the seedlings were randomly placed in three Sealed-top chambers (STC) with four pots in each room. To maintain uniform lighting conditions and eliminate edge effects, the pots in each growth chamber were placed in the center of the control room and randomly located at a circle radius of 0.85 m (the growth chamber radius was 1.7 m). The latest IPCC-predicted temperature and CO_2_ concentration increase was simulated in the first growth room as control check (CK). In one growth chamber, the temperature was increased by 2 °C as a temperature increase treatment (ET), and the CO_2_ concentration of the other growth chamber was set to 850 μmol·mol^−1^ as a CO_2_ concentration doubling treatment (EC); the other environmental factors remained the same. The plants were watered twice a week to maintain uniform flooding depth and growth conditions during the experimental period. The underground-propagule re-grows seedlings in the early spring (March) every year, and the plants were grown for 4 months to mature. Our experiment has carryed out in June 2018.

### Plant functional traits

On sunny days, from 08:30 to 11:30 in the morning, we selected three plants from four pots and measured photosynthesis using a Li-6400 portable photosynthesis instrument (LI-6400, LI-COR, Nebraska, USA). The plants can growth 1 m hight in the chambers. The site of about 20 cm from the top of each leaf is mature enough and has stable structure and function. More importantly, this site can capture enough light, while the lower sites are usually insufficient light. From each plant, two fully developed mature leaves were selected from each plant, arranged them side by side, and put the sites of 20 cm from the top of the two leaves into the chamber at the same time. Then the in situ determination of the net photosynthetic rate (Pn, μmol·m^−2^·s^−1^), stomatal conductance (Gs, mol·m^−2^·s^−1^), and transpiration rate (Tr, mmol·m^−2^·s^−1^) was performed. During the measurement, the light intensity inside the leaf chamber was set to 1500 μmol·m^−2^·s^−1^, the leaf temperature was kept at 22–24 °C, the flow rate was set to 500 μmol·s^−1^, and the indoor CO_2_ mole fraction was set to 425 μmol·s^−1^·mol^−1^.

The leaves used for photosynthesis measurement were cut down to a length of about 15 cm from the middle part, sealed in a bag containing wet paper balls, and stored in a box until analysis of leaf anatomy and determination of hydraulic traits. Part of the leaves was cut, and transparent nail polish was applied on the surface. After drying, the surface was teared off to obtain the surface print, which was placed on a glass slide to observe the stomata of *S. tabernaemontani* under an optical microscope (Leica Inc., DM2500, Bensheim, Germany). Images were taken and processed using the Image J (v. 1.48; http://rsb.info.nih.gov/ij/) image processing software. The number of stomata in each picture was determined and stoma length was measured (SL, μm). Stoma density (SD, No./mm^2^) was calculated as the number of stomata per unit area.

The cross-section of *S. tabernaemontan*i was cut, and thin slices were selected and stained with toluidine blue to prepare water-mounted slices, avoiding the epidermis. In the middle section, pictures of the vascular bundle structure were taken under an optical microscope. The following parameters were measured: vascular bundle length (VBL, μm), vascular bundle width (VBW, μm), vascular bundle area (VBA, μm^2^), and leaf vein density (VD, mm/mm^2^), using the Image J image processing software. Subsequently, the lens was adjusted to the epidermal structure, which was observed under an optical microscope and photographed. Via the Image J image-processing, the following characteristics were measured: cuticle thickness (CT, μm), epidermal thickness (ET, μm), and epidermal cell area (EA, μm^2^). In this study, the repetition amount of each hydraulic trait is 30, and the specific measured traits and their abbreviations are shown in Table 1.

**Table 1:** Functional traits of *Schoenoplectus tabernaemontani* measured in this study and the results of principal components analysis

Vascular bundle traits	Abbreviations	Unit	PC1	PC2
Net photosynthetic rate	P_n_	μmol·m^−2^·s^−1^	0.846**	− 0.224
Stomatal conductance	Gs	mol·m^−2^·s^−1^	0.692*	− 0.396
Transpiration rate	Tr	mmol·m^−2^·s^−1^	0.959**	0.003
Stomatal length	SL	μm	0.698*	-0.576
Stomatal density	SD	no·mm^−2^	− 0.571	0.659*
Vein density	VD	mm·mm^−2^	− 0.587	− 0.159
Cuticle thickness	CT	μm	0.655	− 0.697*
Epidermal thickness	ET	μm	0.530	0.549
Epidermal cell area	EA	μm^2^	0.725*	0.391
Vascular bundle length	VBL	μm	0.921**	0.260
Vascular bundle width	VBW	μm	0.832**	0.462
Vascular bundle area	VBA	μm^2^	0.738*	0.546

*P < 0.05; **P < 0.01

### Data analysis

Using the R (4.0.3) statistical analysis software, and through its built-in "vegan" program package, one-way ANOVA and LSD were applied for multiple comparisons to compare various functional traits of *S.tabernaemontani* under different treatments. The difference test was performed at a statistical significance level was *P* < 0.05. Principal components analysis (PCA) was used to obtain the degree of variation of various functional traits in response to influencing factors. Pearson’s correlation analysis was performed on the functional traits of *S. tabernaemontani* to reveal the synergistic relationships among its functional traits.

## Results

### Responses of *Schoenoplectus tabernaemontani* hydraulic traits to temperature and CO_2_ increases

**Fig. 1:**
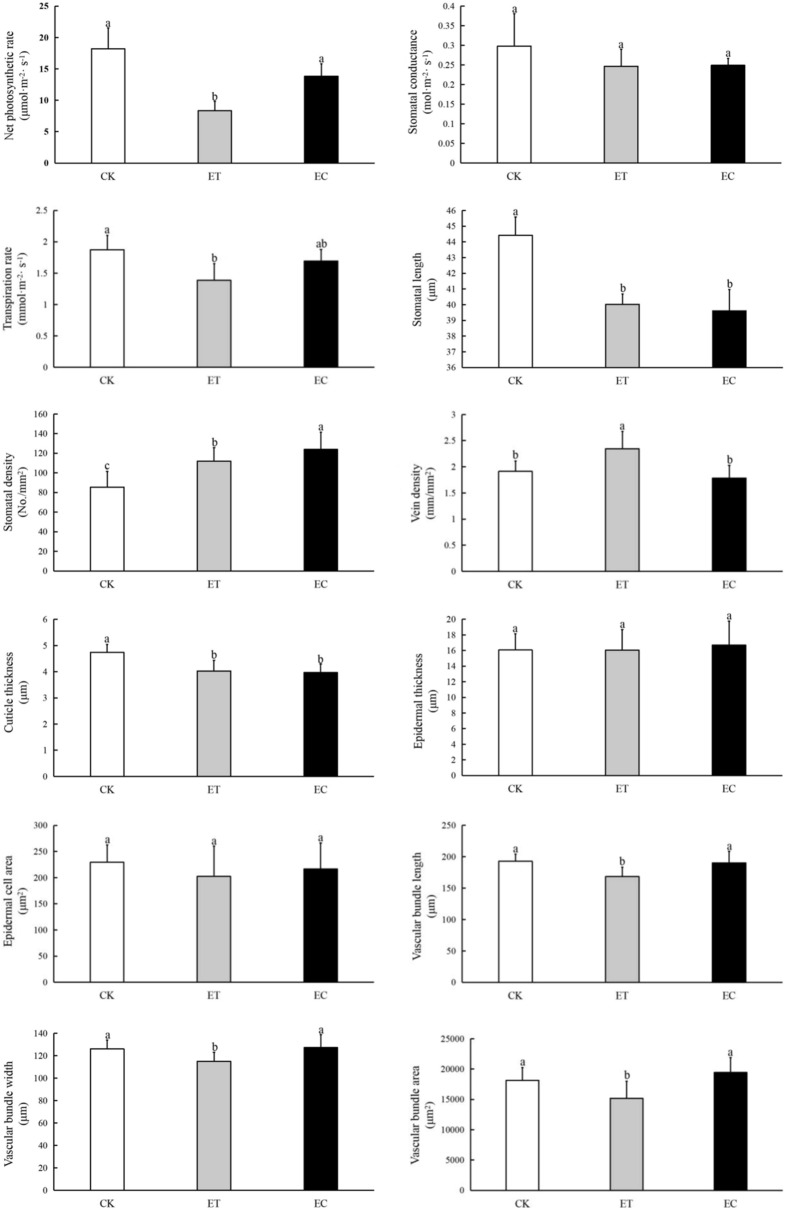
Differences in the hydraulic traits (mean ± SD) of *Schoenoplectus tabernaemontani* among three groups subjected to different treatments. Different lowercase letters indicate significant differences at the 0.05 level (*P* < 0.05). *CK* control group, *ET* warming group, *EC* CO_2_ concentration doubling group

A 2ºC increase in temperature had a more substantial effect on the hydraulic traits of *S. tabernaemontani* than a doubling of the CO_2_ concentration. The temperature increase and CO_2_ concentration doubling treatments generally reduced photosynthetic capacity, stomatal size, and cuticle thickness and increased the stomatal density of *S. tabernaemontani*. However, the response trend of *S. tabernaemontani* hydraulic traits to warming and CO_2_ concentration doubling is not precisely the same (Fig. 1). Compared with the CK group, net photosynthetic rate, transpiration rate, stomatal length, cuticle thickness, vascular bundle length, vascular bundle width, and vascular bundle area of *S. tabernaemontani* in the ET group were significantly reduced, whereas stomatal density and vein density increased significantly. Compared with the CK group, the hydraulic traits of *S. tabernaemontani* in the EC group were reduced considerably in stomatal length and cuticle thickness but increased dramatically in stomatal density, and there were no significant differences between other parameter values and the control group. Compared with the EC group, net photosynthetic rate, stomatal density, and vascular bundle size (including vascular bundle length, width, and area) of *S. tabernaemontani* in the ET group were significantly lower, whereas vein density was substantially higher (Fig. 1).

### Correlation between hydraulic traits of *Schoenoplectus tabernaemontani*

**Fig. 2:**
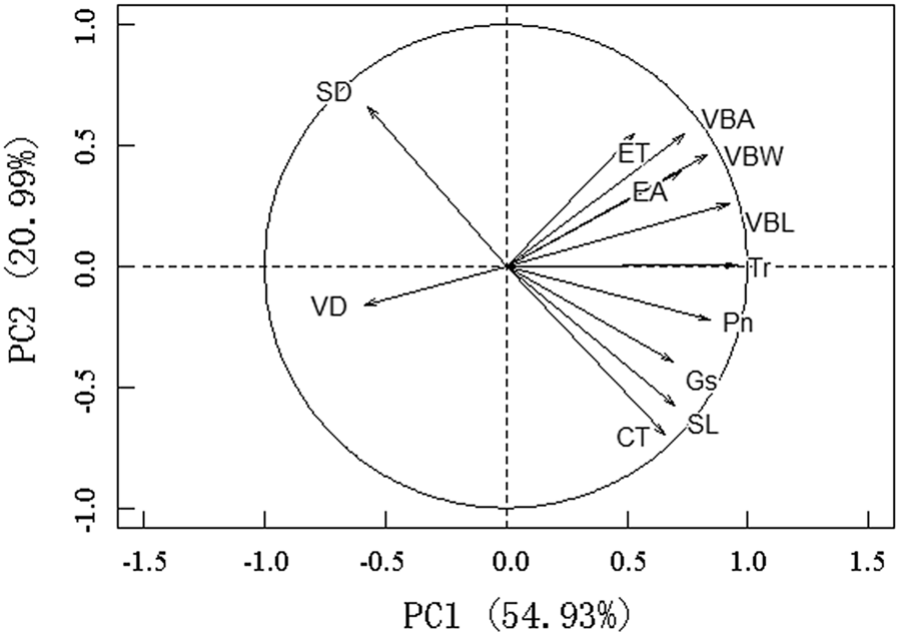
Principal components analysis showing the contribution of hydraulic traits of *Schoenoplectus tabernaemontani* to total variance and the relationships among the characteristics. *Pn* net photosynthetic rate, *Gs* stomatal conductance, *Tr* transpiration rate, *SL* stomatal length, *SD* stomatal density, *VD* vein density, *CT* cuticle thickness, *ET* epidermal thickness, *EA* epidermal cell area, *VBL* vascular bundle length, *VBW* vascular bundle width, *VBA* vascular bundle area

The first two main axes of the principal components analysis based on the hydraulic traits of *S. tabernaemontani* explained 54.93% and 20.99% of the total variation variance of functional traits, respectively (Fig. 2). The first principal axis was significantly positively correlated with net photosynthetic rate, stomatal conductance, transpiration rate, stomatal length, epidermal cell area, vascular bundle length, vascular bundle width, and vascular bundle area. The second principal axis was significantly positively correlated with stomatal density and significantly negatively correlated with cuticle thickness (Table 1).

Net photosynthetic rate was significantly positively correlated with stomatal length, cuticle thickness, and vascular bundle length, and stomatal conductance was significantly positively correlated with cuticle thickness. The transpiration rate was significantly positively correlated with cuticle thickness, epidermal cell area, vascular bundle length, vascular bundle width, and vascular bundle area (Table 2). Regarding the hydraulic traits, there was a significant negative correlation between stomatal density and stomatal length, or cuticle thickness, and a significant positive correlation between the latter two. The epidermal cell area was significantly positively correlated with epidermal thickness, vascular bundle length, vascular bundle width, and vascular bundle area (Table 2).

**Table 2:** Correlations among hydraulic traits of *Schoenoplectus tabernaemontani*

	Pn	Gs	Tr	SL	SD	VD	CT	ET	EA	VBL	VBW
Pn											
Gs	0.629										
Tr	0.888**	0.736*									
SL	0.702*	0.551	0.593								
SD	− 0.445	− 0.622	− 0.425	− 0.864**							
VD	− 0.580	− 0.267	− 0.567	− 0.167	0.127						
CT	0.699*	0.689*	0.660*	0.825**	− 0.776**	− 0.286					
ET	0.183	0.321	0.486	0.110	− 0.090	− 0.093	− 0.099				
EA	0.446	0.449	0.703*	0.365	− 0.235	− 0.063	0.174	0.891**			
VBL	0.666*	0.458	0.844**	0.477	− 0.398	− 0.713*	0.450	0.528	0.655		
VBW	0.582	0.249	0.747*	0.350	− 0.231	− 0.649	0.230	0.592	0.700*	0.956**	
VBA	0.516	0.215	0.695*	0.233	− 0.019	− 0.539	0.133	0.510	0.666*	0.865**	0.901**

*Pn* net photosynthetic rate, *Gs* stomatal conductance, *Tr* transpiration rate, *SL* stomatal length, *SD* stomatal density, *VD* vein density, *CT* cuticle thickness, *ET* epidermal thickness, *EA* epidermal cell area, *VBL* vascular bundle length, *VBW* vascular bundle width, *VBA* vascular bundle area. *P < 0.05; **P < 0.01

## Discussion

Increased temperature and CO_2_ levels are not conducive to the photosynthetic activity of *S. tabernaemontani*. Photosynthetic rate, stomatal density and size, vein density, epidermal structure size, and vascular bundle size play an essential role in the adaptation of this species to changes in temperature and CO_2_ concentration. In the process of adaptation, hydraulic traits are not isolated from each other, and there is a functional association among traits.

Like terrestrial plants, wetland plants show significant changes in hydraulic traits in different climatic environments, reflecting their response strategies. Earlier studies on the ecological responses of wetland plants on the Southwest Plateau of China have shown that at elevated temperatures, *Hippuris vulgaris* increased the aboveground stem vascular structure of the ducts, sieves, and vascular bundles, along with the pronounced development of the belowground vascular network of the ducts and sieves to enhance mechanical supportability and water retention ability (Guan et al. 2019). Similarly, *Schoenoplectus tabernaemontani* significantly reduces its vessel perimeter, area, and cross-sectional surface, the cross-sectional area of the sieve tube, and its net photosynthetic rate but substantially increases the cross-sectional density of the sieve tube to adapt to higher temperatures (Feng et al. 2020a, b). In addition, warming significantly affects the light and CO_2_ use of dominant plants in the wetland lakeside zone of the Northwest Yunnan Plateau, with different species showing different responses. *Zizania latifolia* adapts to warming by reducing its photosynthetic CO_2_ use capacity and net photosynthetic rate (Liu et al. 2017). The species *Sparganium stoloniferum* adapts to warming by increasing its light saturation point, light energy use range, and net photosynthetic rate (Liu et al. 2017). At high CO_2_ concentrations, *S. tabernaemontani* can significantly increase its net photosynthetic rate, intercellular CO_2_ concentration, water use efficiency, and biomass and reduces stomatal conductance and transpiration rate (Xu et al. 2016). By inhibiting the photosynthetic mechanism of the leaves, *Vallisneria natans* lowers its photosynthetic capacity to adapt to changing atmospheric CO_2_ concentrations (Han et al. 2017). These studies reflect the interspecific differences in plateau wetland plants in adapting to increasing temperatures and CO_2_ concentrations, improving our understanding of the functional responses of plateau wetland plants to a changing climate. However, these studies did not comprehensively consider the transportation, loss, and maintenance of water and substances by plants from the hydraulics perspective and neglected the relationship between corresponding traits and photosynthetic production.

Different plant species show different responses to climate warming. Either on a global scale (Wright et al. 2004) or for individual plant species (Yin et al. 2008; Qi et al. 2012; Wang et al. 2017), most studies have shown that the photosynthetic capacity of most plants increases with increasing temperatures, mainly because the increase in temperature promotes the activity of plant photosynthetic enzymes and accelerate the gas exchange rate of plants, thereby promoting photosynthetic activity. Studies on specific types or individual species of plants have found that the relationship between plant photosynthetic capacity and temperature is not significant (Zhao et al. 2016) and decreases with increasing temperatures (Bresson et al. 2011; Liu et al. 2018) or first increases with temperature and then declines (Vo et al. 2015). This reflects the differences in the responses of different plants to temperature changes and indicates that the photosynthetic capacity is not only affected by temperature but also by other environmental factors. Under different environmental conditions and in various ecosystems, there are various controlling factors. For example, plants in high-elevation areas are strongly affected by temperature, light intensity, CO_2_ concentration, and microclimatic conditions (Bresson et al. 2011; Sun et al. 2016a, b). Epiphytes are significantly affected by water availability and light conditions (Sun et al. 2014), whereas wetland plants are generally largely affected by temperature, CO_2_ concentration, water, sediment environment, among others (Zhang et al. 2021). In our study, the photosynthetic and transpiration rates of *S. tabernaemontani* decreased significantly under increasing temperatures (Fig. 1), reflecting the decline in photosynthetic capacity and productivity. This is consistent with the results of Qi et al. (2012) for *Phragmites australis* and the *in-situ* field study by our research team in the Napahai of Shangri-La, Yunnan (Feng et al. 2020a, b), further confirming that against the background of a changing climate, warming is not conducive to photosynthetic production and biomass accumulation of *S. tabernaemontani*. Our earlier field investigations on the plateau area also found that this species is the dominant aquatic-terrestrial ecotone species in the Napahai area at an elevation of 3266 m and an average temperature of 5.4 °C, with its photosynthetic rate exceeding 30 μmol·m^−2^·s^−1^. In contrast, in the Lashihai area at an elevation of 2437 m and an average temperature of 13.6 °C, it is more slender and short and does not dominate the plant community, with a photosynthetic rate only occasionally reaching 20 μmol·m^−2^·s^−1^.

Several studies have found that the responses of herbaceous wetland plants to warming are more complex than those of woody plants. Even for plants colonizing the same habitat, small temperature changes can produce significantly different response trends. Liao et al. (2016) and Wang et al. (2019) have shown that between 1985 and 2008, the temperature in the Napahai has increased by 1.2 °C, and the differential responses of dominant plants in the aquatic-terrestrial ecotone can directly affect the wetland type, distribution area, and landscape diversity. An *in-situ* comparative study on the four plant species *S. tabernaemontani*, *Sparganium emersum*, *H. vulgaris*, and *Eleocharis liouana* in the Napahai of Shangri-La found that compared with the control group, increased temperatures affect the growth of *S. tabernaemontani* and *H. vulgaris* by promoting above-ground stem vascular structure, whereas the development of *E. liouana* and of the underground stem vascular structure of *H. vulgaris* was impeded. Also, the biomass of *S. emersum* first increased and then decreased (Dong et al. 2014; Guan et al. 2018). Plants have a certain tolerance level to changes in temperature. Moderate warming will increase photosynthetic rate, stomatal conductance, transpiration rate, and other parameters that reflect photosynthetic gas exchange capacity, whereas further increases in temperature with impede these processes (Ruan and Li 2001). At present, *S. tabernaemontani* grows in numerous aquatic-terrestrial ecotones on the Yunnan Plateau. It is the dominant plant species in the aquatic-terrestrial ecotone in Shangri-La, Lugu Lake, Dianchi Lake, and other places, indicating that the current temperature in Yunnan is generally suitable for its growth. However, with the predicted further increase in temperature, *S. tabernaemontani* may gradually become less competitive in plateau areas due to its inability to adapt to higher temperatures.

The responses of plant morphological and structural parameters to temperature correspond to the photosynthetic capacity. The stomata are the primary channels for plants to control water vapor exchange, and the greater the density and the smaller the size, the higher the sensitivity of stomatal opening and closing, the higher the rate of water vapor exchange, and the higher the water loss (Franks and Beerling 2009). The vascular structure is the center of water and material transportation and distribution and the main structure to maintain the upright state of plants (Sack et al. 2016; Nelson and Dengler 1997). The greater the vein density, the stronger the conveying capacity, enabling the plant to remain upright and stretched. The larger the vascular bundle structures, the more water, nutrients, and organic matter can be transported by a single vascular bundle, but the risk of cavitation of the vascular bundle is also higher (Chen et al. 2017). Therefore, when plants adapt to environmental stress, those with higher vascular bundle density and smaller tissue structure show increased photosynthetic productivity with transmission efficiency (Sack et al. 2016). On the other hand, plants with low vascular bundles density and larger tissue structure are at risk of vascular bundle cavitation, transporting large amounts of substances simultaneously to increase their photosynthetic production. The leaf epidermis and its appendages provide mechanical support and ultraviolet radiation resistance and prevent physical water loss (Ristic and Jenks 2002). The small and tightly arranged epidermal cells can effectively reduce the water loss rate and maintain the moisture levels in plants (Sun et al. 2016a, b). The cuticle can reduce water evaporation and increase refractivity, preventing plants from damages by intense radiation (Dylan et al. 2009). In this study, the increase in temperature significantly improved stomatal sensitivity and water loss capacity of the studied species while also increasing water and material support via higher vein density (Fig. 1). However, the risk of cavitation blockage of the vascular bundle also increased (larger vascular bundle size), and the physical water retention capacity of the epidermis and the ability to protect the plant against UV damage decreased because of the decreased cuticle thickness. Under warming conditions, higher stomatal density and vein density correspond to lower photosynthetic rates (Table 2). This is consistent with our previous research results for other plateau wetland plants. For example, increasing temperature will reduce the light saturation point, net photosynthetic rate, and other photosynthetic characteristics of *Zizania latifolia*, thereby decreasing the light use ability (Liu et al. 2017). In plateau areas, in addition to transporting water and materials, the vascular structure of wetland plants may also consume a considerable proportion to support the upright stature of plants. This is also related to the fact that wetland plants grow in water and are easily affected by the force of water currents. The significant positive correlation between photosynthetic rate and vascular bundle length (Table 2) indicates that maintaining an upright position of *S. tabernaemontani* is the prerequisite for photosynthetic production. At the same time, the smaller the vascular bundle reduces the risk of cavitation under increasing temperatures. This directly manifests the decline in the photosynthetic capacity of *S. tabernaemontani*.

At increasing temperatures, water loss through the stomata and the epidermis is high, and the photosynthetic activity of *S. tabernaemontani* is considerably more affected by stomatal sensitivity and epidermal water loss (thin cuticle) than by stomatal gas exchange (higher stomatal density). High stomatal water loss and low photosynthetic rate indicate that the water use efficiency of *S. tabernaemontani* is low. The water use efficiency are also decreased in ET and EC conditions (values of CK, ET, and EC are 9.704, 6.008 and 8.168 μmol·mmol^−1^ respectively). In addition to photosynthetic gas exchange, a large part of water is used for other purposes, such as physical cooling of leaves and stomatal opening to obtain more CO_2_. Water loss can be controlled by the physical barriers presented by epidermal structures, such as the cuticle and the epidermis (Kerstiens 1996; Riederer and Schreiber 2001). Greater cuticular thickness is hypothesized to decrease cuticular water permeability and reduce evaporative water loss through the epidermis (Kerstiens 1996; Riederer and Schreiber 2001). The photosynthetic rate was significantly positively correlated with cuticle thickness, indicating that thick cuticle with little water loss through the epidermis may essential for promoting water loss by stomatal gas exchange to increase photosynthetic rate.

The concentration of CO_2_ is closely related to photosynthesis. Since *S. tabernaemontani* is an aquatic plant, it has unlimited access to water; however, in wetland habitats, the amount of available CO_2_ is limited. Under warming conditions, wetland plants may physically cool the leaves with large amounts of readily available water, obtaining limited CO_2_ amounts through the stomata (Zhang et al. 2007). Plants often show enhanced photosynthetic capacity as the CO_2_ concentration rises. However, over time, they adapt to these high concentrations, resulting in a “downregulation of photosynthesis” (Wg 1991; Kimball 1991). In our study, net photosynthetic rate, stomatal conductance, and transpiration rate of *S. tabernaemontani* showed a downward trend under the condition of doubled CO_2_ concentrations but did not reach significant levels. This is consistent with the findings of Jiang et al. (1997), who reported that in some plants, under high CO_2_ concentrations, photosynthesis is downregulated; however, the underlying mechanisms still need to be explored. According to previous studies, elevated CO_2_ concentrations can inhibit photosynthesis via changes in plant physiology and metabolism. Excessive CO_2_ concentrations (> 700 μmol·mol^−1^) in plants will affect the consumption capacity of triose phosphate and the regeneration ability of phosphate radicals in the photophosphorylation process, resulting in a decreased CO_2_ use, which in turn leads to a reduction in the photosynthetic rate (Farquhar 1980). Increasing atmospheric CO_2_ concentrations also increase the intercellular CO_2_ concentrations of plants, and to maintain a stable osmotic potential, plants will adjust the opening and closing of their stomata (Guan et al. 2019; Farquhar and Sharkey 1982).

In our study, *S. tabernaemontani* showed a significant reduction in stomatal length and cuticle thickness under the condition of a doubled CO_2_ concentration, whereas stomatal density was substantially increased. This indicates a trade-off between stomatal and cuticle traits in the adaptation process. Similarly, Liu (2017) showed that the CO_2_ concentration regulates leaf wax synthesis by promoting or inhibiting the expression of leaf wax synthesis regulation genes. Increasing CO_2_ concentrations will significantly reduce leaf wax, which is consistent with the results of this study. Since an increase in CO_2_ reduces the wax synthesis of the leaves of *S. tabernaemontani*, which leads to a significant reduction in cuticle thickness, therefore, water is more likely to be lost through the leaves. The present study found a significant correlation between cuticle thickness and stomatal traits; to maintain its leaf water balance, the *S. tabernaemontani* responded by reducing its stomatal size and increasing its stomatal number, thereby reducing leaf water loss.

## Data Availability

The data that support the findings of this study are available from the corresponding author upon reasonable request.

## Abbreviations

Pn: Net photosynthetic rate
Gs: Stomatal conductance
Tr: Transpiration rate
SL: Stomatal length
SD: Stomatal density
VD: Vein density
CT: Cuticle thickness
ET: Epidermal thickness
EA: Epidermal cell area
VBL: Vascular bundle length
VBW: Vascular bundle width
VBA: Vascular bundle area
CK: Control check
ET: Temperature increase treatment
EC: CO_2_ concentration doubling treatment
CO_2_: Carbon dioxide
*S. tabernaemontani*: *Schoenoplectus tabernaemontani*
